# Targeting high glucose-induced epigenetic modifications at cardiac level: the role of SGLT2 and SGLT2 inhibitors

**DOI:** 10.1186/s12933-023-01754-2

**Published:** 2023-02-02

**Authors:** Lucia Scisciola, Fatemeh Taktaz, Rosaria Anna Fontanella, Ada Pesapane, Surina, Vittoria Cataldo, Puja Ghosh, Martina Franzese, Armando Puocci, Pasquale Paolisso, Concetta Rafaniello, Raffaele Marfella, Maria Rosaria Rizzo, Emanuele Barbato, Marc Vanderheyden, Michelangela Barbieri

**Affiliations:** 1 Department of Advanced Medical and Surgical Sciences, University of Campania “Luigi Vanvitelli”, Naples, Italy; 2 Department of Advanced Biomedical Sciences, University of Naples Federico II, Naples, Italy; 3 Cardiovascular Center Aalst, OLV Hospital, Aalst, Belgium; 4 Department of Experimental Medicine, University of Campania “Luigi Vanvitelli”, Naples, Italy; 5 Mediterranea Cardiocentro, Naples, Italy; 6 Department of Clinical and Molecular Medicine, Sapienza University, Rome, Italy

**Keywords:** SGLT2, SGLT2 inhibitors, Cardiomyocytes, Epigenetic modifications, DNA methylation

## Abstract

**Background:**

Sodium-glucose co-transporters (SGLT) inhibitors (SGLT2i) showed many beneficial effects at the cardiovascular level. Several mechanisms of action have been identified. However, no data on their capability to act via epigenetic mechanisms were reported. Therefore, this study aimed to investigate the ability of SGLT2 inhibitors (SGLT2i) to induce protective effects at the cardiovascular level by acting on DNA methylation.

**Methods:**

To better clarify this issue, the effects of empagliflozin (EMPA) on hyperglycemia-induced epigenetic modifications were evaluated in human ventricular cardiac myoblasts AC16 exposed to hyperglycemia for 7 days. Therefore, the effects of EMPA on DNA methylation of NF-κB, SOD2, and IL-6 genes in AC16 exposed to high glucose were analyzed by pyrosequencing-based methylation analysis. Modifications of gene expression and DNA methylation of NF-κB and SOD2 were confirmed in response to a transient SGLT2 gene silencing in the same cellular model. Moreover, chromatin immunoprecipitation followed by quantitative PCR was performed to evaluate the occupancy of TET2 across the investigated regions of NF-κB and SOD2 promoters.

**Results:**

Seven days of high glucose treatment induced significant demethylation in the promoter regions of NF-kB and SOD2 with a consequent high level in mRNA expression of both genes. The observed DNA demethylation was mediated by increased TET2 expression and binding to the CpGs island in the promoter regions of analyzed genes. Indeed, EMPA prevented the HG-induced demethylation changes by reducing TET2 binding to the investigated promoter region and counteracted the altered gene expression. The transient SGLT2 gene silencing prevented the DNA demethylation observed in promoter regions, thus suggesting a role of SGLT2 as a potential target of the anti-inflammatory and antioxidant effect of EMPA in cardiomyocytes.

**Conclusions:**

In conclusion, our results demonstrated that EMPA, mainly acting on SGLT2, prevented DNA methylation changes induced by high glucose and provided evidence of a new mechanism by which SGLT2i can exert cardio-beneficial effects.

**Graphical Abstract:**

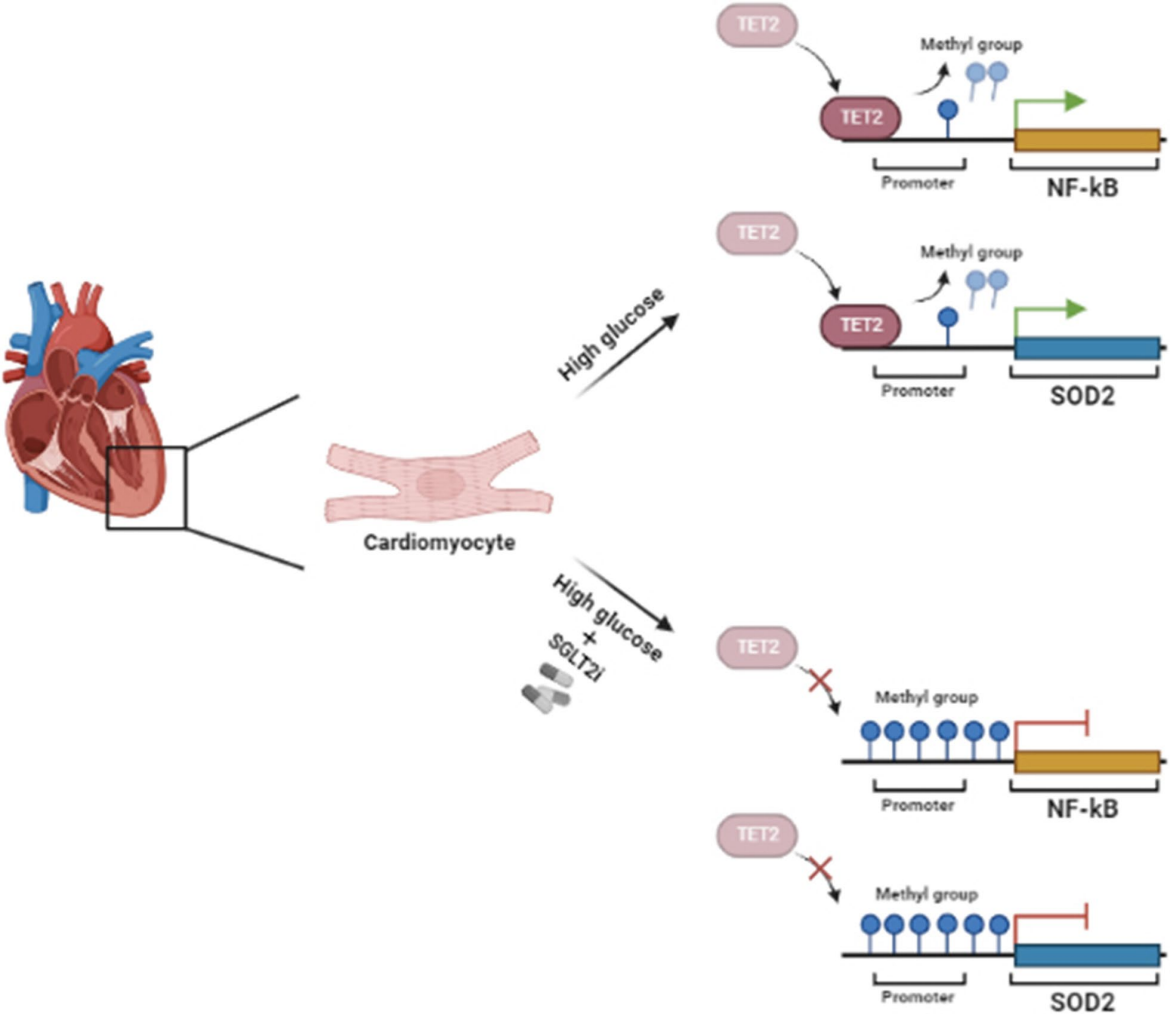

**Supplementary Information:**

The online version contains supplementary material available at 10.1186/s12933-023-01754-2

## Background

Cardiovascular diseases (CVDs) are the leading cause of death globally, with an estimated 17.9 million deaths yearly [1–4]. One of the most important and independent risk factors for heart disease is diabetes mellitus, which predisposes to the development of coronary heart disease (CHD), cerebral vascular disease (CVAs) and/or peripheral arterial disease (PAD) [5–7]. A growing body of evidence demonstrated that epigenetic modifications play an important role in the development and progression of CVD affecting vascular and cardiac function in patients with diabetes [8]. Epigenetics, through DNA methylation, histone modifications, and non-coding RNA regulation, mainly regulates the expression of genes involved in oxidative stress, inflammation, and angiogenesis [9].

In particular, in human aortic endothelial cells (TeloHAEC), high glucose has been found to be associated with significant demethylation in the promoter region of nuclear factor-kB (NF-kB), superoxide dismutase 2 (SOD2), and Sirtuin (SIRT) 6 leading to their detrimental expression. Accordingly, diabetic patients showed a decrease in DNA methylation in the previously mentionedgenes compared to non-diabetic patients [10, 11]. Furthermore, in the atherosclerosis plaque and myocardial ischemia–reperfusion injury model, SIRT1 inhibits NF-kB activity by deacetylating p65 and eliminating the interaction between p300 and NF-kB, reducing Nitric Oxides (NOS) 2 and pro-inflammatory genes [12]. Indeed, the downregulation of SIRT3 expression and its redox inactivation results in SOD2 inactivation promoting the occurrence of hypertension [13].

The sodium-glucose co-transporters inhibitors (SGLTi), a novel class of drugs used to treat patients with type 2 diabetes [14, 15], have become a subject of interest due to their beneficial effects at the cardiovascular level [16–18].

Diverse potential mechanisms of action have been hypothesized and include metabolic and hemodynamic effects as well as effects on inflammation, oxidative stress, and intracellular ion homeostasis [19–21]. Regardless of glucose concentrations, dapagliflozin (DAPA) could exert direct anti-inflammatory effects, at least partly, by inhibiting the expression of Toll-like receptor-4 (TLR-4) and activation of NF-κB along with the secretion of pro-inflammatory mediators [22]. Moreover, SGLT2i attenuated the myocardial mRNA levels of nucleotide-binding domain-like receptor protein 3 (NLRP3) inflammasome targets and the consequent release of pro-inflammatory cytokines, such as cyclooxygenase (COX) 2 and interleukin (IL) 1β in genetic diabetic mice [23–25]. Moreover, (SGLT2i) also act as antioxidant agents by decreasing cardiac oxidative stress and mitochondrial reactive oxygen species (ROS) production [26, 27].

However, the ability of these drugs to induce their cardiac protective effects by acting through epigenetic mechanisms has not been previously investigated.

In the present study, to better elucidate the beneficial properties of SGLT2i in terms of cardiovascular protection, the effects of empagliflozin (EMPA) on hyperglycemia-induced epigenetic modifications were evaluated in human ventricular cardiac myoblasts AC16, exposed to hyperglycemia for 7 days.

## Methods

### Cell culture

AC16 human cardiomyocyte cell lines were purchased from EMD Millipore (cod. SCC109). Following the manufacturer's instructions, the cell line was tested and authenticated for mycoplasma contamination, which was negative. Cells were cultured in Dulbecco's Modified Eagle's Medium (DMEM)/F12 (cod. AL215A, Microgem) containing 12.5% fetal bovine serum (FBS) (cod. ECS0180L, Euroclone), 1% antibiotics penicillin-streptomycin (cod. ECB3001D, Euroclone), and 1% of L-glutamine (cod. ECB3000D, Euroclone). The cell line was maintained in the incubator at 37 °C and 5% CO2. The cells were grown between 5 and 7 passages, and experiments were performed in triplicate. AC16 were exposed to 33 mmol/L D glucose (cod. G8644, EMD Millipore) for 7 days and treated with EMPA at a concentration of 0.5 µM (cod. S8022, BI 10773, Selleckchem) [28]. The medium was changed every 48 h. Normal glucose (NG), considered the control, are cells exposed to normal glucose concentration (5.5 mmol/L) and cultured for 7 days.

### Protein extraction and western blotting

Cells were dissolved in lysis buffer containing protease inhibitors (Tris HCL pH8 10 mM, NaCl 150 mM, NaF 10 mM, NP40 1%, PMSF 1 mM). Then, the proteins were subjected to 10% sodium dodecyl sulfate-polyacrylamide gel electrophoresis (SDS-PAGE) and transferred to 0.22 um polyvinylidene fluoride (PVDF) membranes. The membranes were blocked with 5% non-fat milk in TBS-T (Tris-buffered pH 8 0.15% Tween 20) at room temperature for 1 h and t hen incubated with primary antibodies diluted in TBS-T (dilutions according to datasheet), including antibodies against NF-κB (ab16502, abcam), NF-kB p65 (phospho S276) (ab183559, abcam), Acetyl NF-kB p65 (Lys310) (AF1017, Affbiotech), SOD2 (ab68155, abcam), Acetyl-SOD2 (Lys68) (AF4360, Affbiotech) IL-6 (elab-30095, abcam), SGLT2 (pA5-75567, ThermoFisher Scientific), SGLT1 (ab14686, abcam), overnight at 4 °C. Vinculin (ab129002, abcam) was used for protein expression normalization as an internal control. After three washes in TBS-T, the membrane was incubated with corresponding secondary antibodies, goat anti-rabbit IgG-h + HRP Conjugated (cod. A120-101P Bethyl), for 1 h at room temperature. Immunocomplexes were visualized by using Clarity Max Western ECL Substrate (cat. 1705062, Bio-Rad Laboratories) and visualized by using ChemiDoc Imaging System with Image Lab Software Version 6.1 software (Bio-Rad Laboratories). The molecular weight of proteins was estimated with prestained protein markers (cod. G623 Opti-Protein-Marker abm). Densitometry analysis was performed using Image J software.

### RNA extraction and quantitative real-time PCR

Total RNA was isolated and purified using miRNeasy Mini Kit (cod. 217004, Qiagen) according to the manufacturer's instructions for human cell samples. Then cDNA was synthesized from 1 ug of total RNA using QuantiTect Reverse Transcription Kit (cod. 205310, Qiagen). mRNA levels were determined by qPCR with Green-2 Go qPCR master mix (cod. QPCR004-5 Biobasic) using Rotor-GENE Q (Qiagen).

Primers sequence: NF-κB: fw 5′-AATGGTGGAGTCTGGGAAGG-3′, rv 3′-TCTGAC GTTTCCTCTGCACT-5′; SOD2 fw 5′-AAGTCATCCACCCACCTCAG-3′, rv 5′-CGTGGAGAGAGCATGAAAGC-3′; IL-6: fw 5′-AGTCCTGATCCAGTTCCTGC-3′, 5′- CTACATTTGCCGAAGAGCCC-3′; β-Actin fw 5′-CATCCGCAAAGACCTGTACG-3′, rv 5′-CCTGCTTGC TGATCCACATC-3′

For each amplification cycle, a threshold cycle (C_t_) value was obtained, and Δ_Ct_ was calculated as the C_t_ difference between target mRNA and housekeeping mRNA (β-Actin). The fold increase of mRNA expression compared with NG was calculated using the 2^−ΔΔCt^ method. The histograms reported the genes of 3 separate experiments, where NG value was set as 1.

### Methylation analysis

DNA was extracted using the QIAamp DNA Blood Mini Kit (cod. 51104, Qiagen,) according to the manufacturer's protocols. The methylation analysis of genes was investigated by pyrosequencing-based methylation analysis using the PyroMark Q48 Autoprep (Qiagen) after DNA bisulfite conversion. Bisulfite conversion was performed with 350 μg of DNA isolated using the EpiTect Fast DNA Methylation kit (cod. 59824 Qiagen) as recommended by the manufacturer. The bisulfite-modified DNA was amplified by polymerase chain reaction (PCR) using the PyroMark PCR Kit (cod. 978703 Qiagen). According to the manufacturer's instructions, each reaction mixture contained 2 μl of bisulfite-converted DNA, 12.5 μL of PyroMARK PCR Master Mix 2X, containing Hot Start Tag DNA Polymerase, 2.5 μL of CoralLoad Concentration 10X, and 2.5 μL of mix PCR cycling conditions were 1 cycle at 95 C for 15 min: 40 cycles at 94 °C for 30 s, 56 °C for 30 s, and 72 °C for 10 min. Electrophoresis of the PCR product was performed on a 2% Agarose Gel (Amersham Biosciences). The biotinylate PCR products were subjected to sequencing using a PyroMark Q48 Advanced CpG Reagent (cod. 974022, Qiagen) and analyzed by PyroMark CpG SW 1.0 software (Qiagen). The primers were commercially designed, and codes are listed below: NF-κB: Island n 1 in gene promoter: Hs_NF-κB_01_PM PyroMark CpG assay (PM00110908) bp 103423134 _103423182 CRCh37/hg19, SOD2: Island n 3 in gene promoter: Hs_SOD2_03_PM PyroMark CpG assay (PM00121366) _bp 160114829 _160114864 CRCh37/hg19. For the IL-6 methylation study were used PCR and sequencing custom primers: PCR forward primer (5-AGGGATAATTTAGTTTAGAGTTTATTTGT-3), PCR reverse primer (biotin-5-CTCCCTCTCCCTATA AATCTTAATT-3) and sequencing primer (5-ATAAGAAATTTTTGGGTGT-3).

### Chromatin immunoprecipitation followed by quantitative real-time PCR (ChIP-qPCR)

AC16 cells were cross-linked with 1% of formaldehyde for 20 min at RT, and then cross-linking was stopped by adding 1/7 vol of 1 M glycine. Chromatin samples from 10^6^ cells were sonicated to 200–500 bp in ChIP lysis buffer (20 mM HEPES pH 7.6, 1 mM EDTA, 0.5 mM EGTA, 0.05% SDS, and protease inhibitors). Sonicated chromatin was centrifuged for 10 min and then incubated overnight with 5 μg TET2 antibody (cod. C15410255, Diagenode) and Protein A/G plus (sc-2003 Santa Cruz) in 1X Incubation buffer (50 mM Tris pH 8.0, 750 mM NaCl, 5 mM EDTA, 2.5 mM EGTA, 0.75% SDS, 1% Triton X-100, 0.1% BSA, and protease inhibitors). The following day, sonicated chromatin was washed in buffer 1 (10 mM Tris pH 8.0, 1 mM EDTA, 0,5 mM EGTA, 0.1% DOC, 0.1% SDS, and 0.1% Triton X-100), buffer 2 (10 mM Tris pH 8.0, 1 mM EDTA, 0.5 mM EGTA, 0.1% DOC, 0.1% SDS, 0.1% Triton X-100, and 500 mM NaCl), buffer 3 (10 mM Tris pH 8.0, 1 mM EDTA, 0.5 mM EGTA, 0.25 LiCl, 0.5% DOC, and 0.5% NP40) and buffer 4 (10 mM Tris pH 8.0, 1 mM EDTA, and 0.5 mM EGTA). After the last wash, the supernatant was recovered, and it was eluted in elution buffer (1% SDS and 0.1 M NaHCO3) at RT for 20′- 30′, then, it was incubated ON at 65 °C with 200 mM NaCl. DNA was extracted with phenol: chloroform protocol. ChIP experiments were analyzed by qPCR with specific primers using a Green-2 Go qPCR master mix. Recovery of ChIP and DNAs was calculated as a percentage of IP/Input. The sequences of primer sets used for TET2 were: for NF-κB FW: GCACATGGGATTAGCGACAG, RV: TCCAACCTTCTCACCATCCC; for SOD2 FW: CATGACT GCCAGGGCTTAGT, RV: AGTTCTTGGACACCCACGAC.

### Cell viability assay

Cell viability was assayed by Cell Counting Kit-8 (CCK-8, CK04, Dojindo) according to the manufacturer's protocols. Briefly, AC16 cells were seeded into 96-well plates and treated with high glucose and EMPA for 7 days. After specific treatment, 10 μL of CCK-8 solution was added to each well and incubated for 2 h at 37 °C. The absorbance was then recorded at 450 nm using a microplate reader (Sunrise absorbance reader, TECAN). The relative cell viability was normalized with the control group using optical density values, and three independent experiments were conducted.

### ROS detection: fluorescence-activated cell sorting (FACS)

Intracellular ROS levels were also measured using a ROS detection assay kit (cell-based ab139476). Cells were cultured in 6-well plates and exposed to high glucose and EMPA treatment for 7 days. According to the manufacturer's instructions, cells were collected, washed with 1X wash buffer, and centrifugated for 5 min at 400 × g. According to the kit protocol suggestion, 1 × 10^5^ cells were stained with detection reagent for 1 h at 37 °C in the dark with periodic shaking. Measurements were carried out using BD Accuri C6 Plus Personal Flow Cytometer (BD biosciences) at Ex/Em = 490/525 nm. Data processing was performed using FlowJo BD Accuri C6 Plus software for windows.

### SGLT2 small interfering RNA

AC16 cell lines were transfected with small interfering RNA (siRNA) (30 nM) and with control non-targeting siRNA (NT-siRNA) (30 nM) using RNAiMAX^™^ transfection reagent SGLT2 pool of siRNA consisting of a mixture of three sequences designed for specific human SGLT2. Transfection was performed following the manufacturer's instructions. Briefly, AC16 (1 × 10^5^ cells/well) were seeded in a six wells tissue culture plate 24 h before transfection in an antibiotic-free medium and maintained at 37 °C in 5% CO_2_. After removing the growth medium, the transfection complexes (siRNA-RNAiMAX^™^) were added to the serum-free and antibiotic-free medium. Cells were incubated for 8 h, followed by an additional 16 h of incubation after the addition of FBS (10%) directly to each well, and transfection was performed every 72 h. The cells were treated with high glucose for 7 days. SGLT2 expression was evaluated by Western blot analyses.

### Statistical analysis

Results are reported as the means ± SEM. The difference between the mean values was assessed using a one-way analysis of variance (ANOVA) test. Differences between the mean values were considered significant at a p-value of < 0.05. For real-time, no standard error of mean is reported on control because data are represented as relative measures (fold change) obtained after setting NG equal to 1.

## Results

### Effects of EMPA on cell viability, oxidative stress, and inflammation in cardiomyocytes exposed to high glucose (HG)

**Fig. 1:**
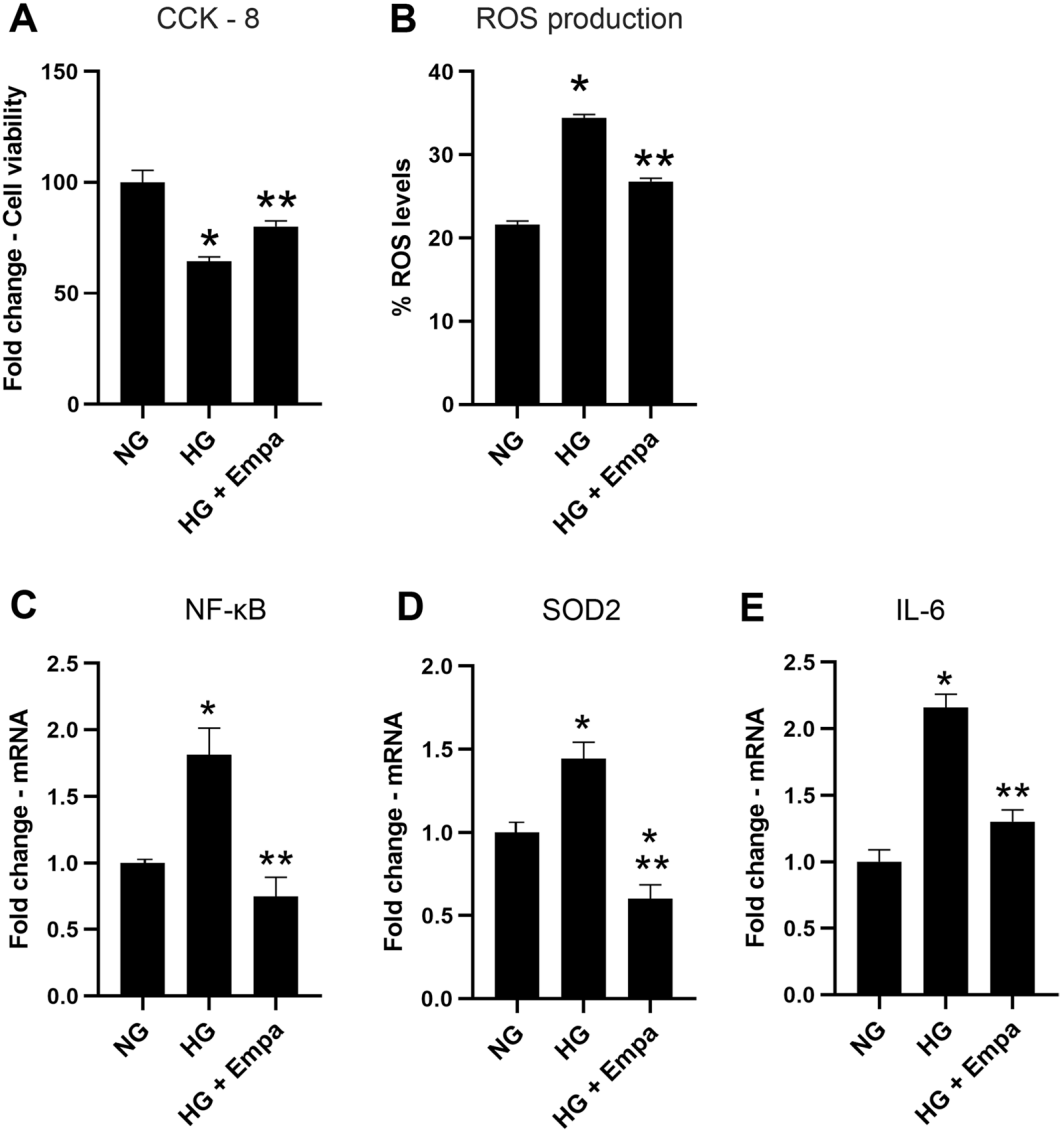
Effects of empa on cell viability, oxidative stress and inflammation in cardiomyocytes exposed to hyperglycemia. **A** Cell viability exanimated using CCK-8 assay and **B**, Intracellular ROS levels, measured using a ROS detection assay kit. **C**–**E**, qRT-PCR for NF-kB, SOD2 and IL-6 in AC16 cells exposed to NG concentration (4.5 mM) (NG), cells exposed to high glucose concentration (HG), and cells co-treated with HG and 0.5 nM of EMPA (HG + Empa). β-Actin was used as internal control. The fold increase of mRNA expression compared with NG was calculated using the 2^−ΔΔCt^ method

Cells exposed to HG for 7 days showed a reduction of cell viability percentage compared to cells exposed to normal glucose (NG) concentration (p < 0.05 vs NG). The co-treatment with EMPA prevented the cell viability reduction induced by HG (p < 0.05 vs HG) (Fig. 1A).

The oxidative stress was evaluated by measuring ROS level. HG 7 days induced an increase in ROS level compared to NG (p < 0.05), an effect counteracted by co-treatment with EMPA (p < 0.05) (Fig. 1B).

mRNA levels and protein concentration of the main genes involved in inflammation and oxidative stress were also quantified.

**Fig. 2:**
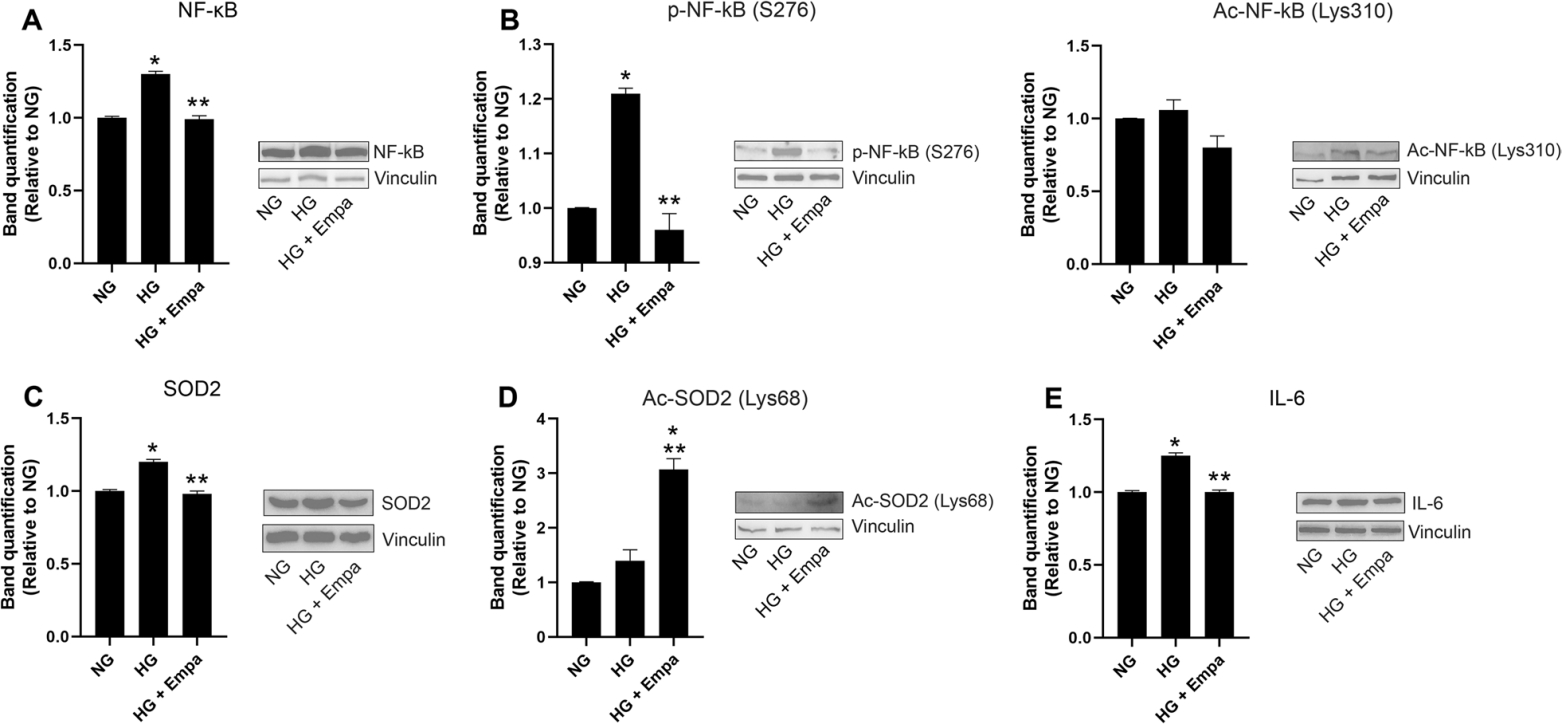
Effects of empa on NF-kB and SOD2 expression and activation. **A** Western blot analysis for acetylated, phosphorylated and total form of NF-kB, and acetylated and total form SOD2 in AC16 cells exposed to NG concentration (4.5 mM) (NG), cells exposed to high glucose concentration (HG), and cells co-treated with HG and 0.5 nM of EMPA (HG + Empa). Densitometry analysis was performed using ImageJ 1.52n software. The histograms show the densitometric analysis of 3 separate experiments representing the relative expression being NG value set as 1. Data are mean ± SEM. * P < vs NG; ** P < vs HG 7 days

HG induced an increment in NF-κB, SOD2, and IL-6 mRNA expression levels compared to the NG condition (p < 0.05). A statistically significant reduction in these markers was observed in cells co-treated with EMPA (p < 0.05) (Fig. 1C, D, E). The same modulation was observed for protein concentration (Fig. 2A, C, E).

Moreover, the phosphorylated form of NF-kB (S276) and acetylated forms of NF-kB (Lys310) and SOD2 (Lys68) were measured to evaluate the effects of EMPA treatment on their activation. Specifically, HG increased the phosphorylated form of NF-kB compared to NG (p < 0.05), suggesting its activation. A statistically significant reduction of phosphor-NF-kB (Lys310) was observed in cells co-treated with EMPA compared to HG (p < 0.05) (Fig. 2B). Conversely, no differences were observed in acetylated form of NF-kB (Fig. 2B) in HG and NG treated cells.

For SOD2 activation, no differences were found in acetylation levels of SOD2 in HG compared to NG. However, the co-treatment with EMPA induced an up-regulation in Acetyl-SOD2(Lys68) suggesting its inhibition (p < 0.05) (Fig. 2D).

### Effects of EMPA on DNA methylation of NF-κB, SOD2, and IL-6 genes in cardiomyocytes exposed to high glucose

**Fig. 3:**
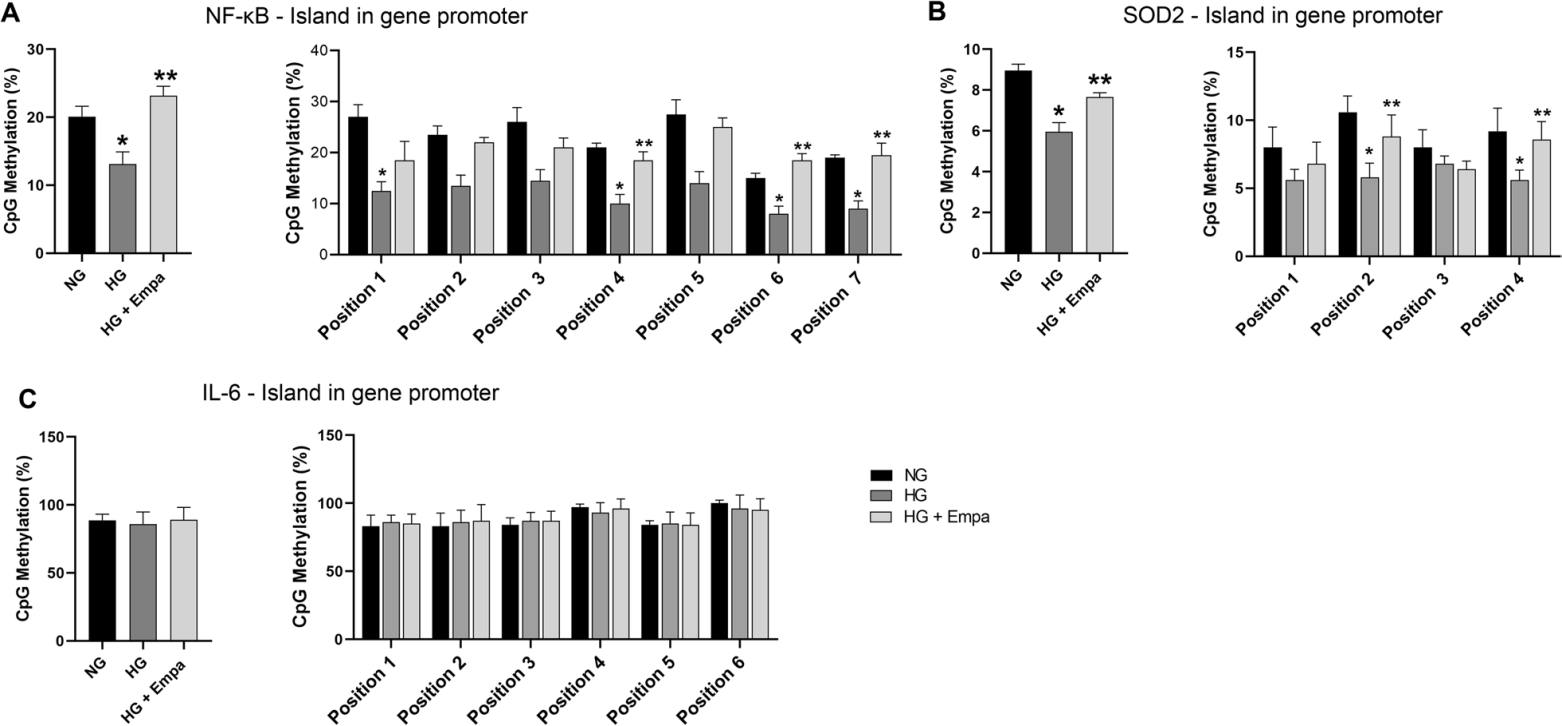
Effects Of Empa On Dna Methylation In NF-kB, SOD2 and IL-6 promoter region in cardiomyocytes. **A**–**C**, DNA methylation analysis of NF-kB, SOD2 and IL-6 promoters expressed as percentage of CpGs methylation. Data are mean ± SEM. * P < vs NG; ** P < vs HG 7 days

Cells exposed to HG showed lower total DNA methylation levels in the NF-κB promoter region compared to cells exposed to NG concentration (p < 0.05). Co-treatment with EMPA prevented the demethylation induced by HG (p < 0.05) (Fig. 3A).

By analyzing, individually, all the positions studied, HG-induced a reduction in methylation levels in four out of the positions analyzed, with a significant effect in positions 1, 4, 6, 7 (p < 0.05 vs NG). At the same time, EMPA significantly prevented the HG-induced reduction in methylation level in positions 4, 6, and 7 (p < 0.05) (Fig. 3A).

The methylation level was also examined in the promoter region of SOD2. Cells exposed to HG showed lower levels of DNA methylation in the investigated island compared to NG (p < 0.05), while EMPA counteracted the observed hypomethylation induced by HG (p < 0.05) (Fig. 3B). The analysis of each position revealed that HG induced demethylation with significant effect in positions number 2 and 4 (p < 0.05 vs NG), and in the same positions, EMPA prevented the demethylation (p < 0.05) (Fig. 3B). No difference between NG, HG, and co-treatment with EMPA was found in the DNA methylation level of the IL-6 promoter region (Fig. 3C).

### Effects of EMPA on DNMTs and ten-eleven translocation (TETs) enzymes

The effects of EMPA on the mRNA expression of the main enzymes involved in DNA methylation were investigated in AC16 exposed to HG for 7 days.

**Fig. 4:**
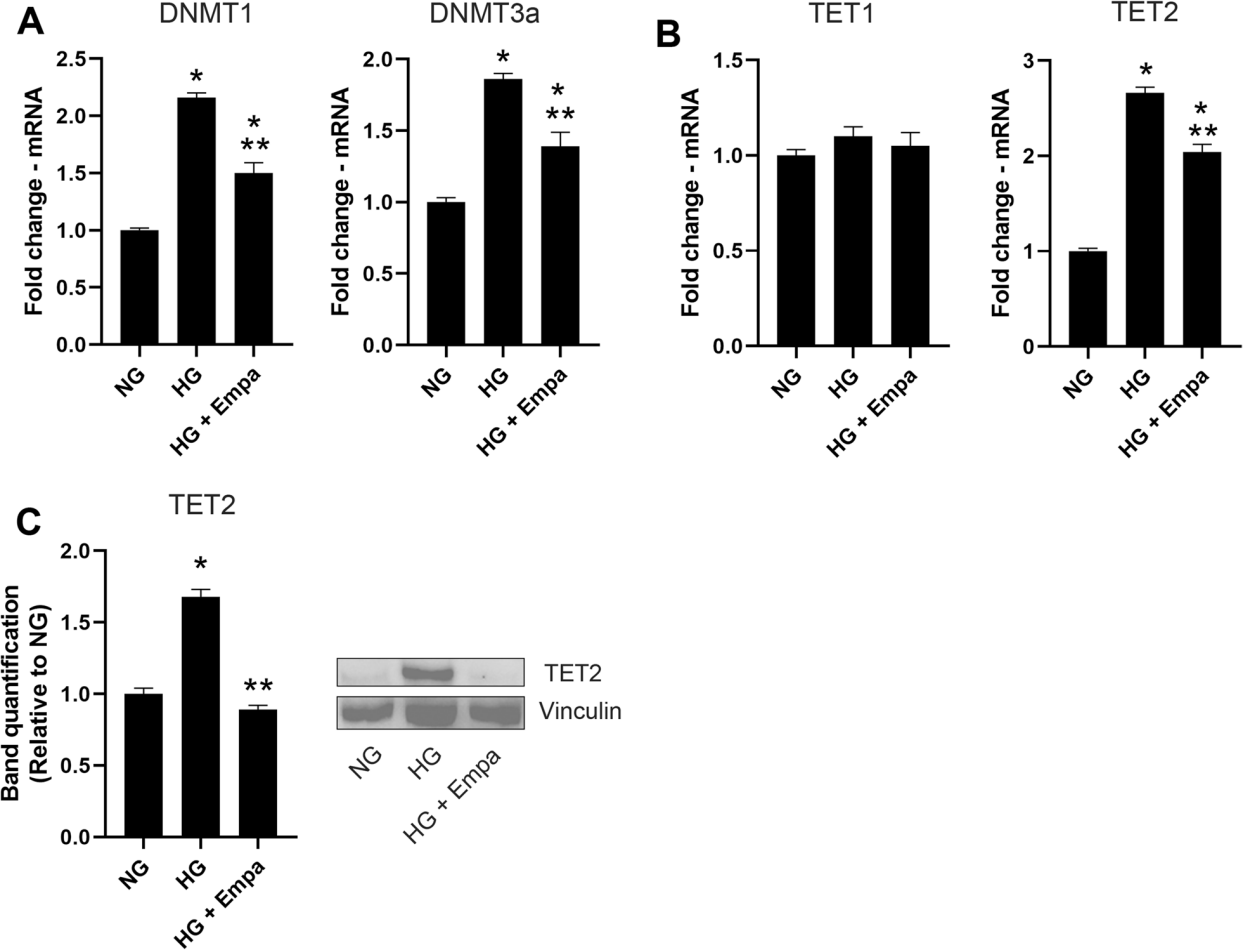
Effects of empa on DNMTs and TETs enzymes. **A**–**B**, qRT-PCR for DNMT1, DNMT3a, TET1 and TET2. C, western blot for TET2 in AC16 cell lines in response to treatment with NG, HG and HG + EMPA. β-Actin was used as internal control. The fold increase of mRNA expression compared with NG was calculated using the 2^−ΔΔCt^ method. Densitometry analysis was performed using ImageJ 1.52n software. The histograms show the densitometric analysis of 3 separate experiments representing the relative expression being NG value set as 1. Data are mean ± SEM. * P < vs NG; ** P < vs HG 7 days.

HG induced the up-regulation of mRNA expression of DNA methyltransferases, DNMT1, and DNMT3a, compared to cells exposed to NG concentration (p < 0.05) (Fig. 4A). The co-treatment with EMPA induced a reduction in DNMT1 and DNMT3a compared to HG (p < 0.05) (Fig. 4A).

No differences were observed in TET1 enzyme expression (Fig. 4B). At mRNA levels, an up-regulation of TET2 was observed in cells exposed to high glucose for 7 days compared to NG (p < 0.05), whereas the treatment with EMPA reduced the HG-induced TET2 increment (p < 0.05) (Fig. 4B). The same modulation was also observed for TET2 protein concentration (Fig. 4C).

To verify a direct link between HG, EMPA treatment, TET2 regulation, and DNA methylation in NF-κB and SOD2 promoters ChIP-qPCR was performed to evaluate the occupancy of TET2 across the investigated regions.

**Fig. 5:**
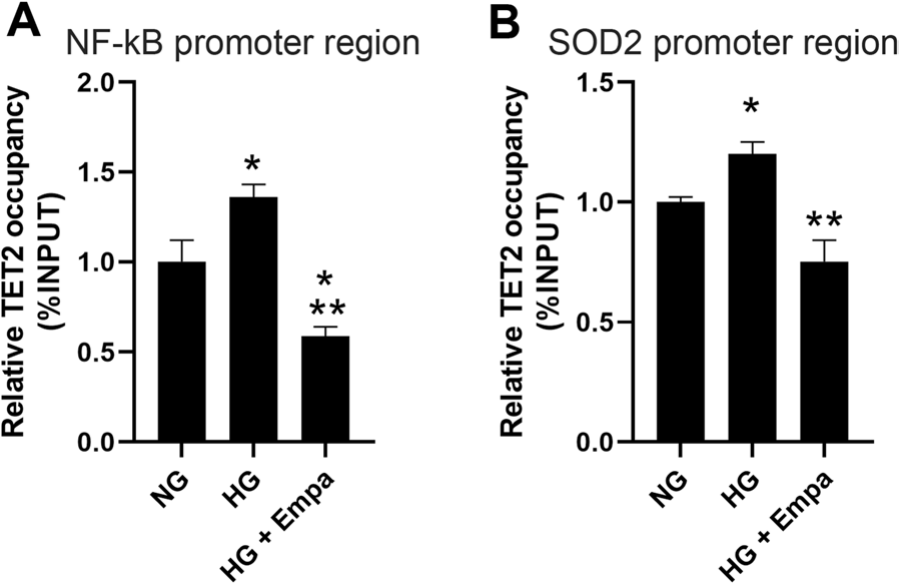
TET2 recruitment on NF-kB and SOD2 promoter region. A, B chromatin immunoprecipitation followed by qPCR. Recovery on ChIP was calculated as percentage of IP/Input. Data are mean ± SEM. * P < vs NG; ** P < vs HG 7 days

In AC16 cells exposed to HG, TET2 binding to CpG island in NF-κB and SOD2 promoter region was higher compared to cells exposed to NG concentration (p < 0.05) (Fig. 5A). Co-treatment with EMPA reduced the TET2 occupancy compared to cells exposed to HG concentration (p < 0.05) (Fig. 5B).

### mRNA and DNA methylation of NF-κB and SOD2 in transient SGLT2 gene silencing exposed to HG for 7 days

Transient SGLT2 gene silencing was performed to understand if SGLT2 mediated epigenetic regulation induced by EMPA. To this end, knockdown of SGLT2 was obtained by treating AC16 cells with a specific SGLT2 RNA interference every 72 h. These cells were treated for 7 days with high glucose and EMPA.

HG-SGLT2 silenced AC16 cells showed a significant reduction in SGLT2 expression levels (p < 0.05), whereas no difference in SGLT1 expression was observed (Additional file 1: Fig. S1).

mRNA and DNA methylation levels of NF-κB and SOD2 were evaluated in HG-SGLT2-silenced cells with and without EMPA.

**Fig. 6:**
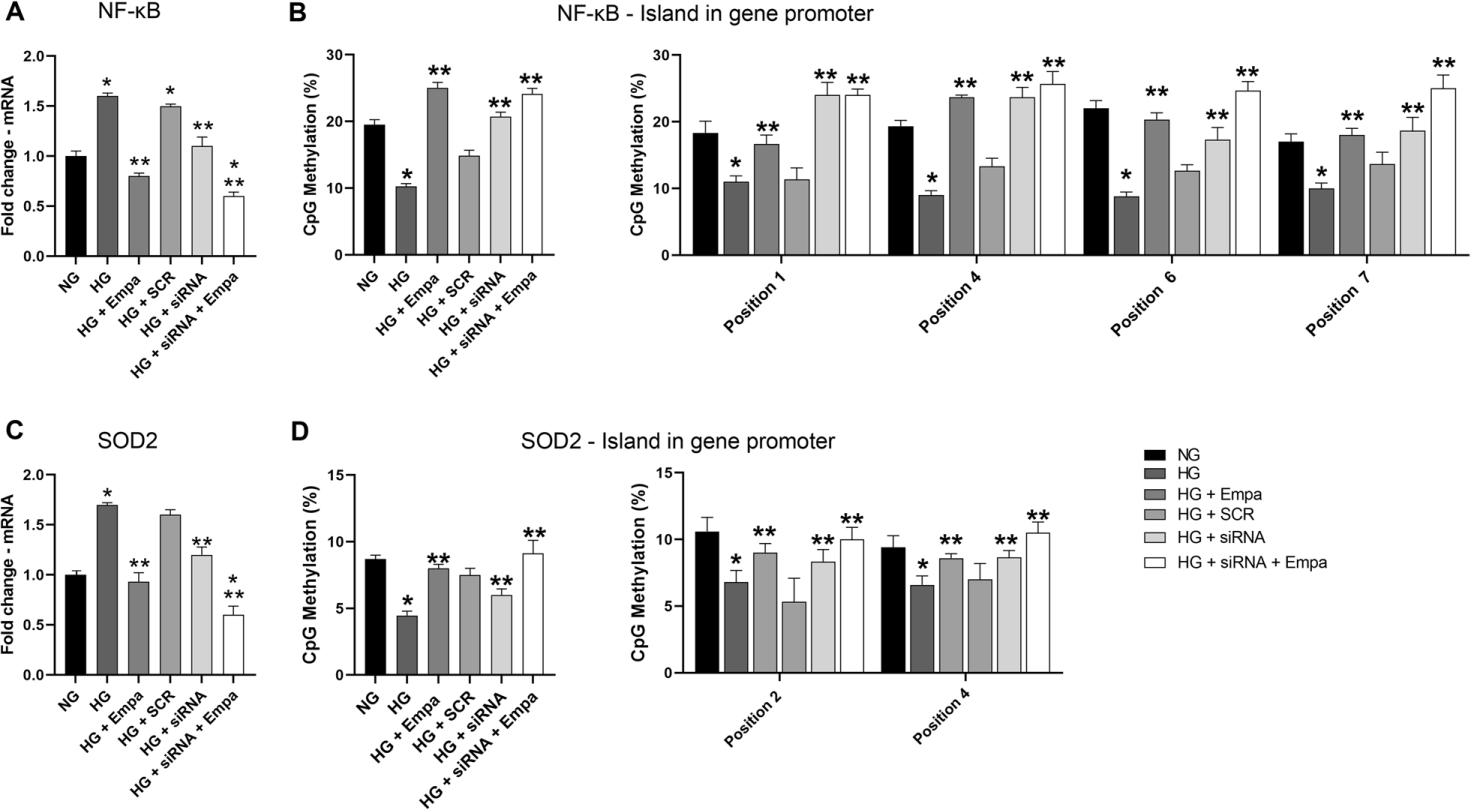
Gene expression and dna methylation of NF-kB AND SOD2 in transient SGLT2 gene silencing. **A**, **B** qPCR for NF-kB and SOD2 in SGLT2 siRNA-transfected AC16 cells exposed to glucose concentration for 7 days. NG = cells exposed to NG concentration, HG = cells exposed to HG concentration for 7 days, HG + EMPA = cells exposed to HG concentration and treated with EMPA; HG + SCR = scrambled siRNA-transfected cells exposed for 7 days to HG; HG + siRNA = SGLT2 siRNA-transfected AC16 cells exposed to HG concentration for 7 days; and HG + siRNA + EMPA = GLT2 siRNA-transfected AC16 cells exposed to 33 mM glucose concentration for 7 days. Data are mean ± SD. * P < 0.05 vs CTR; ** P < 0.05 vs HG.

In particular, HG-SGLT2 silenced cells showed lower NF-κB mRNA expression levels compared to HG 7 days (p < 0.05) but higher levels than HG + EMPA (Fig. 6A). No differences in NF-κB mRNA levels between HG + EMPA cells and HG-SGLT2 silenced cells + EMPA were found (Fig. 6A).

Accordingly, the total DNA methylation level in the NF-κB gene promoter was higher in HG-SGLT2 silenced cells compared to HG (p < 0.05) but lower than HG + EMPA (Fig. 6B). No statistical difference was found between HG + EMPA cells and HG-SGLT2 silenced cells + EMPA (Fig. 6B).

By analyzing, individually, all the positions studied, the HG-SGLT2 knockdown with and without EMPA prevented, with a significant effect, the demethylation in positions 1, 4, 6, 7 (p < 0.05 vs NG) (Fig. 6B).

As far as SOD2 DNA Methylation and expression, a significant reduction of SOD2 mRNA levels was also found in HG-SGLT2 silenced cells compared to HG (p < 0.05), but lower than the reduction observed in HG + EMPA cells (Fig. 6C). Accordingly, HG-SGLT2 silenced cells showed higher total DNA methylation levels compared to HG (p < 0.05) but lower than HG + EMPA (Fig. 6D) or HG-SGLT2 silenced cells + EMPA (Fig. 6D).

Analyzing each position, the SGLT2 silencing with or without EMPA treatment increased DNA methylation with significant effects in position number 2 and 4 (p < 0.05 vs HG) (Fig. 6D).

## Discussion

Our study demonstrates that i) in human AC16 cells, high glucose treatment induces significant demethylation in the promoter regions of NF-kB and SOD2; ii) the observed DNA demethylation is mediated by an increase of TET2 binding to the CpGs island in NF-kB and SOD2 promoters; iii) EMPA prevents HG-induced demethylation changes by reducing TET2 binding to the investigated promoter region and counteracts the altered genes expression; iv) transient SGLT2 gene silencing prevents the DNA demethylation observed in promoter regions thus suggesting a role of SGLT2 as a potential target of the anti-inflammatory and antioxidant effect of EMPA in cardiomyocytes.

Hyperglycemia, the main pathogenetic mechanism of diabetes, induces changes in redox status, inflammation, metabolic profiles, intracellular signaling pathways, and energy production, predisposing to cardiovascular diseases [29].

Preclinical models and human studies have addressed the link between epigenetic factors, type 2 diabetes, and cardiovascular diseases. Hyperglycemia induces epigenetic changes that lead to the altered expression of genes implicated in oxidative stress and inflammation [30]. It was demonstrated that high glucose correlates with a modified DNA methylation pattern [31].

In a zebrafish model of diabetes induced by streptozotocin injection, hyperglycemia induced a 5–tenfold increase in RNA expression of TETs through poly (ADP-ribose) polymerases (PARP) activation [32, 33]. Indeed, to corroborate such an association, the administration of PARP inhibitors prevented the increment in 5hmC induced by hyperglycemia, suggesting a role in TETs regulation [32].

Moreover, our previous studies demonstrated that in human aortic endothelial cells, high glucose induced an increase in TET2 binding on NF-kB and SIRT6 promoter regions, leading to significant demethylation and, consequently, an increase in gene expression. In agreement, also diabetic patients showed statistically significant lower levels of NF-κB and SIRT6 DNA methylation compared to non-diabetic patients [10, 11].

In addition, SET7, a lysin methyltransferase, in response to a change in glucose concentration, translocates in the nucleus regulating the NF-kB pathway [34, 35]. Moreover, hyperglycemia reduced H3K4me1 and -me2 and increased the binding of Lysine-specific demethylase 1 (LSD1) and Sp1 at the Sod2 gene [36].

Interestingly enough, our results demonstrate that, also in human cardiomyocytes, high glucose exposure induces an increase in NF-κB and SOD2 expression through an increment in the demethylation levels of specific CpGs islands located in their promoter regions, which might affect cardiac function and be associated with the development and progression of cardiovascular disease.

The simultaneous activation of the epigenetic machinery and the increased binding of TET2 in the promoter region of the investigated gene demonstrates that glucose exposure, DNA methylation, and gene expression changes are causally linked. More intriguing, our results first showed that treatment with EMPA reduced the expression levels and TET2 binding to the promoter region of NF-kB and SOD2, preventing the HG-induced demethylation and restoring the normal levels of gene expression.

SGLT2i antidiabetic class demonstrates large cardiovascular benefits in diabetic and non-diabetic patients mainly due to systemic effects derived from glycemic control that improve metabolic, hormonal, and hemodynamic whole-body homeostatic [37, 38]. However, additional mechanisms due to direct effects on cardiac cells, such as effects on inflammation, oxidative stress, and intracellular ion homeostasis, were also identified [23]. In particular, it has been demonstrated, in High-Fat Diet–Induced Obese Mice, that EMPA improved myocardial hypertrophy/fibrosis and cardiac function and reduced cardiac fat accumulation and mitochondrial injury, augmenting Sestrin2 levels and increasing AMPK and endothelial nitric oxide synthase phosphorylation [39].

Furthermore, EMPA ameliorates sunitinib-induced cardiac dysfunction by regulating cardiomyocyte autophagy, which was mediated by the AMPK-mTOR signaling pathway in C57BL/6 J mice treated with sunitinib and EMPA [40].

In this regard, recent studies demonstrated that SGLT2i reduced cardiac inflammation through the inhibition of cardiac NLRP3 inflammasome [24, 41], and the reduction of the levels of myocardial pro-inflammatory cytokines, including apoptosis-associated speck-like protein (ASC) containing a caspase recruitment domain (CARD), caspase-1, IL-1β, IL-6 and tumor necrosis factorα (TNFα) [41, 42]. Furthermore, SGLT2i has been shown to also play an essential role in the reduction of oxidative stress, which is a primary contributor to the pathogenesis of the cardiovascular disease. Moreover, EMPA also affected the acetylation and phosphorylation status of NF-kB and SOD2, showing its capability to act at transcriptional and post-transduction levels [22, 43–45].

Nishitani S et al. showed that DAPA-treated mice had higher circulating and tissue levels of β-hydroxybutyrate, a molecule involved in histone modification [46] and speculated that the beneficial health effects of SGLT2i could be associated with epigenetic mechanism. Indeed, any convincing data supporting their hypothesis were provided.

Furthermore, Solini et al. demonstrated that DAPA modulates miRNA expression. Indeed, by upregulating miRNA-30e-5p, DAPA inhibits myocardiocyte autophagy and heart failure, and by reducing miRNA-199a-3p, DAPA causes a reduction in cardiac Peroxisome proliferator-receptor (PPAR) levels, ameliorating mitochondrial fatty acid oxidation and improving cardiac function in patients with heart failure. Moreover, dapagliflozin exerts nephroprotection by preserving renal vasodilating capacity by reducing miRNA-27b expression [47]. These results first suggested epigenetic mechanisms of SGLT2i in improving cardiac functions through miRNA modulations.

Our data not only confirmed the effects of SGLT2i on NF-KB acetylation and phosphorylation previously demonstrated in kidney tissue, HUVEC, macrophages, and H9c2 [22, 43–46], but for the first time, evidence their ability to exert anti-inflammatory and antioxidant effect by modulating NF-kB and SOD2 DNA methylation, directly targeting cardiomyocyte SGLT2.

Previous studies demonstrated that SGLT2i effects at the cardiac level are mediated through the modulations of SGLT1, Na + /H + exchanger 1 (NHE1), Ca2 + /calmodulin-dependent protein kinase II (CaMKII), and late Na + current (late INa) [48]. Indeed, we recently provided evidence that SGLT2 protein is expressed in the human hearts of diabetic and non-diabetic patients and human cardiomyocyte and that hyperglycemia condition induces its overexpression. In addition, the observed high glucose induced cardiomyocyte SGLT2 overexpression is associated with increased oxidative stress, inflammation, and apoptosis, which in turn leads to heart dysfunction. More intriguing, the silencing of SGLT2 blunted mitochondrial oxidative proteins cyclooxygenase (COX) -IV, Cytochrome c and increased the expression levels of the guardian SIRT3 in cardiomyocytes exposed to high glucose [49]. Moreover, SIRT1 and SIRT6 were identified as crucial co-regulators of the SGLT2 network in a bioinformatics analysis evaluating autophagy, oxidative stress, aging, senescence, inflammation, AMPK pathways, and mTOR pathways [50].

Therefore, in light of such recent evidence, it cannot be ruled out that SGLT2i might prevent the HG-induced demethylation and expression changes observed in NF-kB and SOD genes by acting through direct inhibition of SGLT2. To verify such hypothesis, the effect of EMPA, an SGLT2i with the greatest selectivity for SGLT2 [51], on epigenetic machinery was tested in cardiomyocytes exposed to high glucose conditions and treated with small SGLT2 RNA interfering. Interestingly, our results showed that the SGLT2 silencing prevented the HG-induced hypo-methylation in the promoter region of NF-kb and SOD2 without any significant differences with cells treated only with HG + EMPA. These results confirm the hypothesis that the interaction with SGLT2 might mainly explain the observed epigenetic effects of SGLT2 inhibitors. In addition, the more substantial effect observed with a double inhibition treatment (EMPA + SGLT2 silencing) also suggests that other targets, ie. SGLT1, Na channel might be probably also involved.

### Potential in vivo implications

Our study showing an "in vitro" causal link between SGLT2 inhibition, DNA methylation, and gene expression changes demonstrates that SGLT2 inhibitors are potential therapeutic epigenetic regulators, thus suggesting a possible clinical implication of our results.

An "in vivo" detrimental cardiac effect of hyperglycemia on DNA methylation has been previously demonstrated in diabetic patients [10, 11, 52]. More specifically, plasma glucose levels were found to be negatively correlated with DNA methylation in peripheral leukocytes of the promoter region of NF-kB genes. Interestingly enough, in diabetic patients, oral hypoglycemic agent therapy resulted in a significant predictor of NF-κB DNA methylation, independently of age, sex, body mass index, glucose, and plasma lipid levels [10, 11]. Furthermore, a significant correlation analysis of DNA methylation profiles with intima-media thickness, a surrogate marker for early atherosclerosis, left ventricular mass, left ventricular ejection fraction, and cardiac performance index was also found in 365 healthy subjects independently of the other risk factors [52].

We acknowledge that results obtained only in vitro is a potential limitation of our study and that further in vivo studies are necessary for validating our data. Notwithstanding, this previous evidence strongly suggests the potential clinical implication of our results in terms of cardiovascular outcome. Furthermore, results showing that both EMPA and SGLT2 silencing reduced the expression levels and TET2 binding to the promoter region of NF-kB and SOD2, preventing the HG-induced demethylation and restoring the normal levels of gene expression, clearly demonstrate that SGLT 2 inhibition, DNA methylation and gene expression changes are causally linked.

## Conclusions

In conclusion, our results demonstrated that EMPA, mainly acting on SGLT2, prevented DNA methylation changes induced by high glucose and provided evidence of a new mechanism by which SGLT2i can exert cardio-beneficial effects. However, further studies will be necessary to go deeper in the understanding of the mechanisms underlining the SGLT2i action, also taking into account another different target of SGLT2i at cardiac levels.

## Supplementary Information

**Additional file 1: Figure S1.** SGLT2 and SGLT1 protein expression levels in non-transfected AC16 cells, non-transfected AC16 cells exposed to HG for 7 days, scrambled siRNA-transfected cells and SGLT2 siRNA-transfected cells exposed to HG for 7 days.

## Data Availability

The data used and/or analyzed during the current study are available from the corresponding author on reasonable request.

## Abbreviations

ASC: Apoptosis-associated speck-like protein
CaMKII: Ca2 + /calmodulin-dependent protein kinase II
CARD: Caspase recruitment domain
CHD: Coronary heart disease
COX: Cyclooxygenase
CVAs: Cerebral vascular disease
CVDs: Cardiovascular diseases
DAPA: Dapagliflozin
EMPA: Empagliflozin
ILs: Interleukins
late INa: Late Na + current
LSD1: Lysine-specific demethylase 1
NF-kB: Nuclear factor-kB
NHE1: Na + /H + exchanger 1
NLRP3: Nucleotide-binding domain-like receptor protein 3
NOS: Nitric oxides
PAD: Peripheral arterial disease
PARP: Poly (ADP-ribose) polymerase
PPAR: Peroxysome proliferator-receptor
ROS: Reactive oxygen species
SGLTi: Sodium-glucose co-transporters inhibitors
SIRTs: Sirtuins
SOD2: Superoxide dismutase 2
TeloHAEC: Human aortic endothelial cells
TETs: Ten-eleven translocation
TLR-4: Toll-like receptor-4
TNFα: Tumor necrosis factor α
