# Supplementary material for: Targeting high glucose-induced epigenetic modifications at cardiac level: the role of SGLT2 and SGLT2 inhibitors
Source: Cardiovasc Diabetol. 2023 Feb 2;22:24. doi: 10.1186/s12933-023-01754-2 (PMC9896756; doi:10.1186/s12933-023-01754-2)
Supplement: Supplementary file 1 — Additional file 1: Figure S1. SGLT2 and SGLT1 protein expression levels in non-transfected AC16 cells, non-transfected AC16 cells exposed to HG for 7 days, scrambled siRNA-transfected cells and SGLT2 siRNA-transfected cells exposed to HG for 7 days. [file 12933_2023_1754_MOESM1_ESM.docx]

**Additional File 1**

**TARGETING HIGH GLUCOSE-INDUsssCED EPIGENETIC MODIFICATIONS AT CARDIAC LEVELS: THE ROLE OF SGLT2 AND SGLT2 INHIBITORS**

**^‡^**Lucia Scisciola^a^, **^‡^**Fatemeh Taktaz^a^, Rosaria Anna Fontanella^a^, Ada Pesapane^a^, Surina^a^, Vittoria Cataldo^a^, Puja Ghosh^a^, Martina Franzese^a^, Armando Puocci^a^, Pasquale Paolisso ^b,c^, Concetta Rafaniello^d^, Raffaele Marfella^a, e^, Maria Rosaria Rizzo^a^, Emanuele Barbato^f^, Marc Vanderheyden^c^, Michelangela Barbieri^a^

**^‡^Co-first authorship**

^a^Department of Advanced Medical and Surgical Sciences, University of Campania "Luigi Vanvitelli", Naples, Italy; ^b^ Department of Advanced Biomedical Sciences, University of Naples Federico II, Naples, Italy; ^c^ Cardiovascular Center Aalst, OLV Hospital, Aalst, Belgium; ^d^ Department of Experimental Medicine, University of Campania “Luigi Vanvitelli”, Naples, Italy; ^e^ Mediterranea Cardiocentro, Napoli, Italy; ^f^ Department of Clinical and Molecular Medicine, Sapienza University, Rome, Italy

**Brief title:** SGLT2 inhibitors and myocardial epigenetic modification

**Address correspondence to:**

Michelangela Barbieri, MD

Tel: ++390815665138

Fax: ++390815665143

e-mail: [michelangela.barbieri@unicampania.it](mailto:michelangela.barbieri@unicampania.it)

**
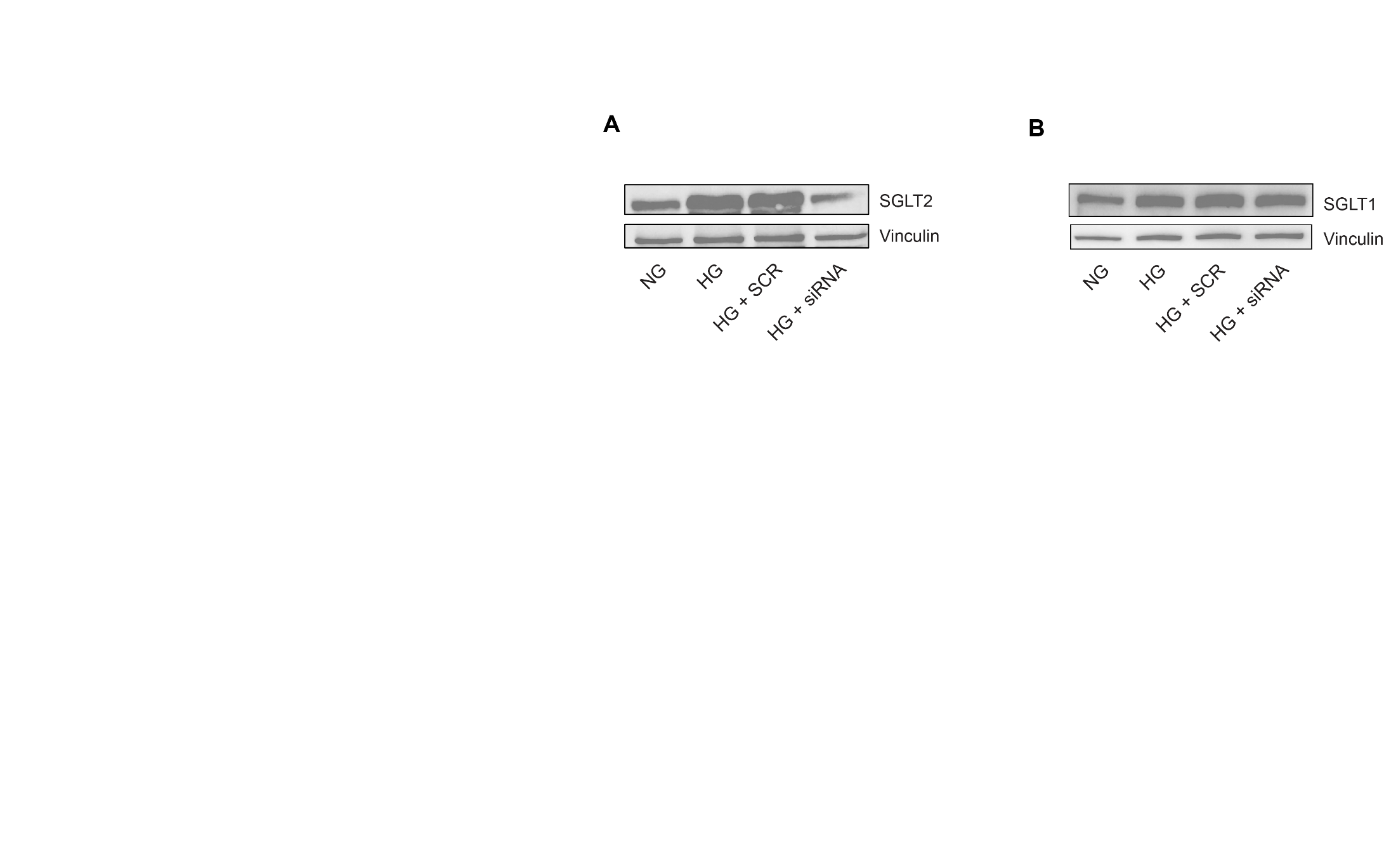
**

**Supplemental Figure 1:** SGLT2 and SGLT1 protein expression levels in non-transfected AC16 cells, non-transfected AC16 cells exposed to HG for 7 days, scrambled siRNA-transfected cells and SGLT2 siRNA-transfected cells exposed to HG for 7 days.
